# COVID-19 Lockdown in New Zealand: Perceived Stress and Wellbeing among International Health Students Who Were Essential Frontline Workers

**DOI:** 10.3390/ijerph19159688

**Published:** 2022-08-06

**Authors:** Anita Jagroop-Dearing, Griffin Leonard, Syed M. Shahid, Ondene van Dulm

**Affiliations:** 1 School of Health and Sport Science, Eastern Institute of Technology, Taradale Campus, 501 Gloucester Street, Hawkes Bay 4112, New Zealand; 2 Kōhatu Centre for Hauora Māori, Otago Medical School, University of Otago, Frederick Street, Dunedin 9016, New Zealand; 3 School of Health and Sport Science, Eastern Institute of Technology, Auckland Campus, 238 Queen Street, Auckland 1010, New Zealand

**Keywords:** COVID-19, perceived stress, wellbeing, international nursing and health students, essential frontline workers, New Zealand

## Abstract

This study examined the stresses and wellbeing of international postgraduate health and nursing students at a tertiary education institute in New Zealand who were mainly essential frontline healthcare workers during the COVID-19 lockdown. Quantitative and qualitative data were collected by purposeful sampling (*n* = 43). The study utilised a cross-sectional survey, along with the Short Form of Cohen’s Perceived Stress Scale (PSS-10), adapted for the COVID-19 lockdown, and followed by semi-structured individual interviews. This study is the first in New Zealand to demonstrate that, with a mean PSS-10 score of 21.7 (±7.1), international health students experienced higher than optimal levels of stress, with supporting qualitative data identifying four themes for the sources of stress: (1) familial relationships, (2) essential work, (3) finances, and (4) study. However, these students coped because of the extensive support provided by their education institute and employers. These students played a critical role in the pandemic’s response and made a significant public health contribution by working in the frontline of the COVID-19 outbreak. Considering the global shortage of healthcare workers and understanding the key challenges, means of coping and support provisions, as we have here, offer insights for building and maintaining a resilient and resourceful health workforce through international health and nursing students in New Zealand and elsewhere.

## 1. Introduction

The COVID-19 virus has spread across the globe, causing the most detrimental pandemic of the last century. The World Health Organisation (WHO) officially determined the COVID-19 outbreak to be a pandemic on 11 March 2020, and globally, it has infected more than 565 million people and caused more than 6 million deaths worldwide [1]. The spread of the virus and public health measures taken to combat its spread have radically changed international travel, workplace practices, and people’s ability to gather socially [2]. An ever-growing body of literature aims to understand the consequences of the COVID-19 pandemic, both in terms of health and wellbeing [3,4] (Dhama et al., 2020; Szcześniak et al., 2021) and the restrictions imposed in order to reduce or eliminate it [5,6]. Globally, there was a shortage of nurses and essential healthcare workers who, at the peak of the outbreak, became new heroes [7].

New Zealand was considered a shining example to the rest of the world in the way the COVID-19 outbreak was managed. We adopted a ‘go hard and go early’ approach and was one of the first countries to introduce a comprehensive nationwide alert level system. An early lockdown with efficient health messaging, effective track-and-trace methods, and the closure of all borders led to the elimination of COVID-19 at the milestone of 100 days after the last community transmission was identified on 1 May 2020 [8]. However, a significant public health concern was the lack of nurses and healthcare workers, which became even more apparent since New Zealand is largely reliant on foreign skilled health workers who make up a staggering 40% of the health work force [7,9].

The first confirmed case of COVID-19 in New Zealand was announced on 28 February 2020, which instigated New Zealand’s Response to COVID-19 (The timeline presented herein is based on that produced by the Ministry of Health, New Zealand https://www.health.govt.nz/our-work/diseases-and-conditions/COVID-19-novel-coronavirus, accessed on 28 January 2022). This study focuses on the COVID-19 lockdown that was initiated 25 March 2020 and remained in place at various alert levels over the following year. Alert levels ranged from 1 to 4 (AL1-4), with 4 being the most restrictive. All non-essential industries, along with travel and physical interaction with those outside one’s household, were prohibited. Border closures had a significant effect on New Zealanders and visitors in the country at the time. One such visitor population comprised international postgraduate nursing and health science students (PG NHSc) who traditionally come to New Zealand to benefit from its world-renowned education system, coupled with a sought-after lifestyle [10]. They proceed to make important academic and economic contributions to their host-institutions and societies [11]. Lockdown restrictions were relaxed in some parts of New Zealand after the first wave of COVID-19 lockdown (AL4), which lasted about six weeks. However, stringent measures of lockdown continued to be applied on a regional basis depending on the COVID-19 community transmission of that region. Thus, our PG NHSc students remained in lockdown (at the various alert levels) for nearly one year, as all courses were transitioned to online learning to facilitate isolation and combating the COVID-19 virus.

We sought to understand the impacts of New Zealand’s lockdown on international PG NHSc at a tertiary education institute, the Eastern Institute of Technology (EIT). In this study, students’ wellbeing was considered in terms of the stress they experienced, the challenges they faced, and their coping mechanisms. This cohort of students is of particular interest because their background in health science and nursing meant that many were classified as ‘essential health workers’ and were, therefore, called upon to work in New Zealand’s healthcare and essential services during the lockdown, while continuing with postgraduate study. NZ immigration normally allows postgraduate students to work up to 20 h per week, but during the COVID-19 lockdown, this was waved with no limitations on the number of hours that students worked. Prior to COVID-19, although some students might work in the healthcare sector, they would generally choose from a range of sectors; this basically included any jobs that were available and brought in extra money while studying towards their postgraduate qualifications. These jobs included roles such as childcare, cleaning, clothes retail, restaurants, etc. Not only do international students make positive contributions to their local communities, amongst other inputs, they also fill the gaps in skilled labour shortages and pre-COVID-19 contribute an astonishing USD 3 billion to the economy annually, with an average expenditure of USD 21,000 per student on fees, living costs, travel, etc. [12]. It is therefore worth understanding the stress, challenges, and associated coping mechanisms of international students in order to best support this population’s health and wellbeing as the global effects of the COVID-19 pandemic continue with ever emerging ‘new’ variants of the coronavirus. In addition, we should reflect on lessons learnt from this experience in preparation for potential similar events in the future.

New Zealand’s time at AL4 was one of strict restrictions imposed on people’s lives. Work, travel, exercise, socialisation, and religious gatherings, among other things, were affected. Research globally has begun to demonstrate the impact of such circumstances on such things as mental health, sleeping patterns, alcohol consumption, and productivity [13,14]. In particular, it has been found that such circumstances are associated with higher levels of stress [15,16], which is a focus in this study. A number of studies have produced data pertaining to the levels of perceived stress in certain populations, some focusing on populations more similar to the population of interest in this study than others. Data presented by Cohen and Janicki-Deverts (2012) offer some of the broadest findings, suggesting that, during 2009 for those living in the United States, the mean PSS-10 scores were 16.14 for women and 15.52 for men [17]. As it pertains to university students, Kwok and Ng [18] found that students between the ages of 18 and 29 years in Hong Kong had a mean PSS-10 score of 19.02, while health science students in Saudi Arabia were found to have a higher mean PSS-10 score of 28.5 [19]. In contrast to this, international postgraduate students in Malaysia were found to have a mean moderate PSS-10 score of 18.19 [20]. There is a rapidly developing body of literature considering the impacts of the COVID-19 pandemic on levels of perceived stress, both among national populations and university students [21,22]. During the pandemic, Turkish university students have been found to have average PSS-10 scores of 23.88 and 21.26 for women and men, respectively [23]. Moreover, the average PSS-10 scores among Swiss university students during April 2020 were found to be less than 15 [24], indicating moderate perceived stress levels. During the COVID-19 pandemic, general populations in Colombia, Hungary, and Paraguay were found to have mean PSS-10 scores of 16.5, 19.34, and 18.10, respectively [25,26,27]. It is noteworthy that some of these studies employed a variant of the PSS-10 known as the PSS-10-C, which seeks to elicit information specifically concerning perceived stress associated with COVID-19 [28]. The findings of these studies have differed in terms of whether variables such as gender have a statistically significant relationship with the PSS-10 score. It seems reasonable to assume that lockdown conditions may have produced unique challenges for New Zealand’s international PG NHSc students and, more generally, among international students worldwide. There are a number of reasons for this: International students already face the challenge of navigating an unfamiliar culture and location while completing their studies, often without the support of a pre-existing social network within the country in which they are studying [29,30]. The challenges of such circumstances were exacerbated by the restrictions imposed as a result of the COVID-19 pandemic. For example, families from students’ country of origin who had planned to join them were no longer able to do so because of closed borders to non-New Zealand citizens. Similarly, students were unable to return to their country of origin to support family, some of whom were in countries that suffered far more severe impacts from COVID-19 than New Zealand. Furthermore, the postgraduate health science students were already qualified health professionals so that many worked extra hours as essential workers during the lockdown, potentially allowing for the continuation of some social contact but also increasing study and work pressure and exposure to potential infection with the virus.

The value of investigating the experiences of international PG NHSc students during New Zealand’s lockdown lies partly in providing these students an opportunity to express their concerns and tell their stories in this unprecedented and challenging time. We also provide information on how to support them in continuing both their study and work in similar future scenarios. Finally, some findings may also be relevant to the global population of international students.

## 2. Methods

This study utilised a cross-sectional survey design and semi-structured interviews to investigate the experiences and psychological stresses among international PG NHSc students who were front-line health workers over the first COVID-19 lockdown period in New Zealand. The online survey contained a combination of select-choice questions and free-text entry questions. The survey was compiled in Google Forms and distributed to international students enrolled in EIT’s Postgraduate Diploma of Health Science, Master of Health Science and Master of Nursing Science programmes from two campuses (Auckland and Hawkes Bay) at EIT. For the purpose of this study, all students enrolled in these programmes are referred as health science students.

An online survey of approximately 20 min was conducted between 21 September and 26 October 2020 while the international student campuses were still in lockdown. The survey collected (i) demographic information, (ii) qualitative data on challenges faced and coping strategies and achievements throughout the lockdown, and (iii) quantitative data on perceived stress levels, the latter by utilising Cohen’s Short Form of Perceived Stress Scale (PSS-10) [31] adapted for the COVID-19 lockdown event [25,28,32]. Cohen’s PSS-10 instrument enjoys wide and enduring usage as an instrument for measuring perceived stress levels and reports on situations of daily life, which are considered as stressful (in this research specified as lockdown and isolation due to the outbreak of COVID-19). Ten questions were asked about participants’ feelings and thoughts over the specified lockdown period (see Table 1). Answers were scored as follows: 0—never; 1—almost never; 2—sometimes; 3—fairly often; and 4—very often. Participant total scores on the PSS-10 can range from 0 to 40, with higher scores indicating higher perceived stress (see results below in the Descriptive Statistics of Perceived Levels of Stress) [17,23,32,33].

Qualitative data were subjected to an inductive, semantic thematic analysis guided by the principles outlined by Braun and Clarke [34]. Thus, additional qualitative data were gathered by semi-structured interviews conducted by the principal investigator via ZOOM video conferencing technology, with open-ended questions where participants were able to speak freely beyond the online survey questions. Qualitative data descriptions allowed for a rich, textural description of students’ experiences in their own words [35,36]. The interviews lasted between 30 and 45 min and were audio-recorded and transcribed verbatim. All transcriptions were checked by at least two researchers to ensure accuracy. The Braun Clarke Thematic Analysis [34] method and the strategy defined by Miles and Huberman [37] were used in this qualitative data analysis process. This approach articulates patterns and themes related to various clinical phenomena to help create an understanding of participants’ experiences [38]. Two reliable researchers (AJ-D and S.M.S) repeatedly read the interview records and scrutinised all lines so that they became familiar with the contents of the data. The two researchers highlighted phrases, sentences, and passages in the transcripts as an initial step to generating inductive codes. The wider team discussed the preliminary codes in detail and devised a coding scheme that was applied to all transcribed data. The resulting codes were extracted and examined by the research team for a second time. After in-depth discussions, relevant codes were grouped into categories depending on similarities and were then conceptually organised into themes and sub-themes. The trustworthiness of the results was ensured by the investigator triangulation method [39].

The study adhered to the ethical requirements pertaining to voluntariness, informed consent, confidentiality, and the anonymity of participation. Ethical approval for this research was obtained from Eastern Institute of Technology Research Ethics and Approvals Committee (REAC approval number 20/20).

### Statistical Analysis

Statistical analysis was carried out using IBM SPSS (Statistical Package for Social Sciences) version 28. For each of the Perceived Stress Scale (PSS-10) questions asked, a frequency and percentage were obtained. Perceived severity was provided in median with interquartile range and the mean ± SD was provided for quantitative variables of PSS-10 scores. The normality of data was checked using the Shapiro–Wilk test in which there was a normal distribution of data. Thus, *t*-tests were used when appropriate (if there were matched number groups). A *p*-value < 0.05 was considered as significant. Univariate and adjusted odds ratios (OR) with 95% confidence intervals (95% CI) were assessed. The reliability and the validity of the PSS-10 was determined by Cronbach’s alpha to be 0.86.

## 3. Results

### 3.1. Demographic Data

Purposive sampling was carried out. All 51 international students enrolled in the above programmes during New Zealand’s lockdown were invited to participate in this study. Forty-three (84%) responded to the survey. Data pertaining to the PSS-10 are derived from 42 respondents. Thirty-eight (88%) participants were female; five (12%) were male. Nineteen (44%) participants were aged 18–29 years, twenty-three (53%) were aged 30–44 years, and one participant was aged 45–54 years, where the mean age was 31.3 ± 7.3. Participants’ countries of origin included India (32 participants, 74%), the Philippines (*n* = 6, 14%), Sri Lanka (*n* = 3, 8%), Nepal (*n* = 1, 2%), and Brazil (*n* = 1, 2%). Thirty-nine (91%) participants had left their families in their home country. Twenty-nine (67%) participants worked as essential health workers in aged care facilities, hospitals, and pharmacies.

### 3.2. Descriptive Statistics of Perceived Level of Stress

**Figure 1:**
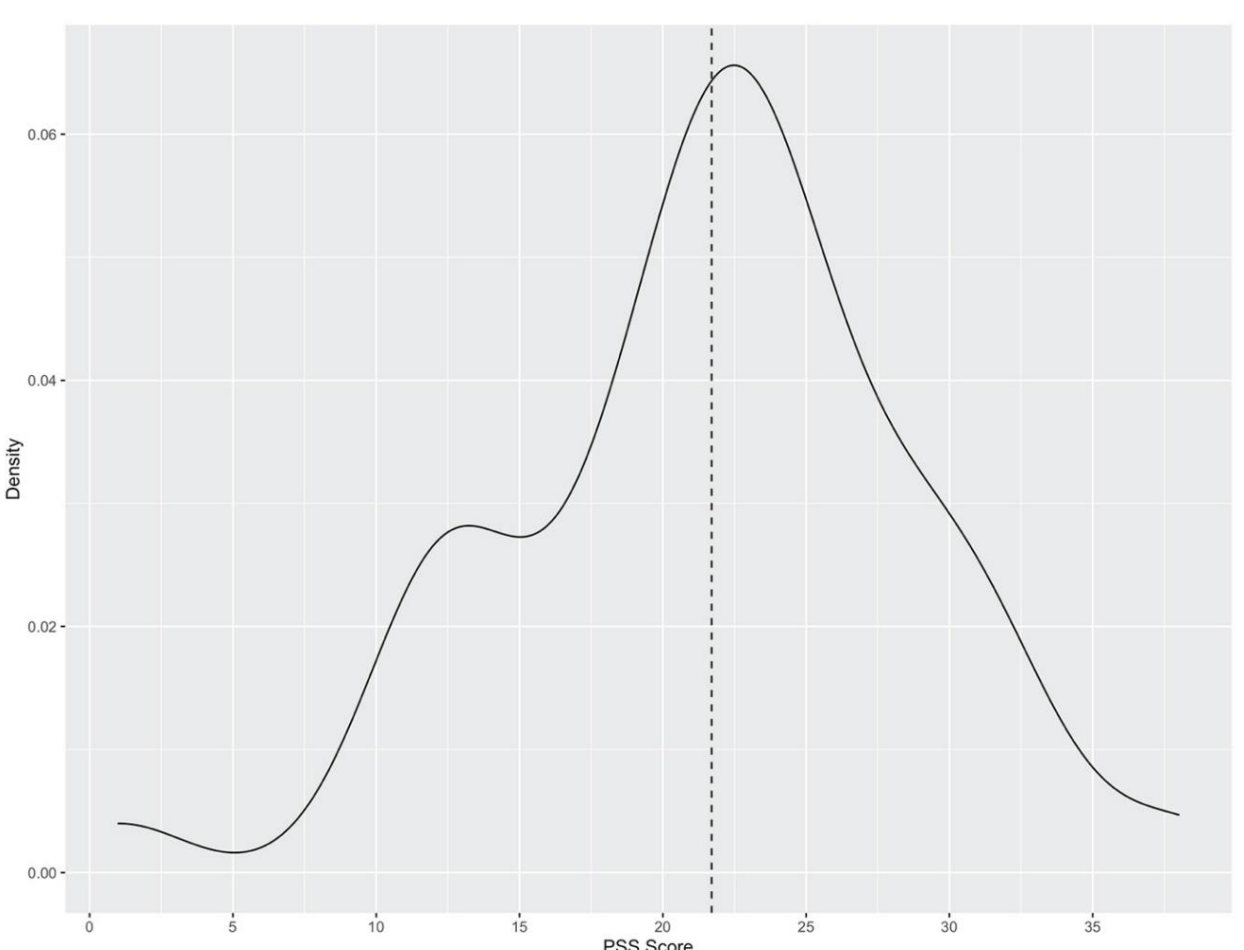
Distribution of Perceived Stress Scores (PSS-10) among participants (observations, *n* = 42); mean ± SD of PSS-10 = 21.7 (±7.1).

The PSS-10 (COVID-19 adapted) required participants to respond to 10 items designed to tap how unpredictable, uncontrollable, and overloaded respondents found their lives over the time of interest. The scale also included a number of direct queries about current levels of experienced stress [40]. Key features of the data collected in response to the PSS-10 scale in the present survey showed that, with 42 observations, the PSS-10 ranged from 1 to 38 with a mean ± SD of 21.7 (±7.1). These are visually depicted in Figure 1. If, as discussed above, non-responses to the survey occurred in a random manner, a sample size of 42, relative to the population size of 51, provides a margin of error of seven percent, where the confidence level is 95%. In other words, we can expect that, 95 times out of 100, a sample of a similar size drawn from the population of interest (those enrolled in the aforementioned courses) would similarly report a mean PSS-10 score of 21.7, plus or minus seven percent. This is to say that a PSS-10 score between 20.2 and 23.2 was observed.

On average, of the 42 participants who responded to the PSS-10 survey, those who performed essential work (*n* = 28, 67%) had lower PSS-10 scores than those who did not (*n* = 14, 33%) perform essential work (PSS-10 = 20.4 compared to 24.3, respectively), although this difference was not statistically significant. Abstract categories of what values constitute low, medium, or high levels of perceived stress do not exist, as the PSS-10 is not a diagnostic instrument. However, scores from 0 to 13 may be considered to reflect low perceived stress, 14 to 26 for moderate perceived stress, and 27 to 40 for high perceived stress [17,40,41]. The findings in the present study can be contextualised by reviewing normative data outlining average levels of perceived stress within other populations (as described in the introduction), although direct comparisons cannot be made due to the non-diagnostic nature of the PSS-10, as well as differences in the populations of interest and research design across studies.

In this study, statistical comparisons in PSS-10 scores between male and female or amongst the various age groups of these participants were not carried out due to unmatched participant numbers for these categories.

Given the context reflected by the findings above, we believe that the mean PSS-10 score of 21.7 (±7.1) in the present study, when viewed in conjunction with the qualitative data discussed below, indicates a higher than optimal level of stress among the population of interest. The PSS-10 scores of the present sample generally exceed or are comparable to scores among both national populations and university students elsewhere during the COVID-19 pandemic [42]. Given the circumstances the present participants (and much of New Zealand and the world) were experiencing during the lockdown, we do not regard a high degree of stress as surprising, nor do we wish to argue that the present sample was either more or less stressed than any other specific group. Rather, we simply suggest that a mean PSS-10 score of 21.7 indicates a higher-than-optimal degree of perceived stress, that this may well affect wellbeing and productivity, and that additional support might be required.

### 3.3. Qualitative Data

The qualitative analysis offers insight into sources of perceived stress, coping measures utilised, general wellbeing, and some outcomes achieved during the COVID-19 lockdown amongst the participants. Table 2 provides the semi-structured guided questions relating to the impacts of the COVID-19 pandemic and associated lockdown on various aspects of participants’ lives, including familial relationships, study, workplace practices, and finances.

A thematic approach was used to identify the emergent themes [34,43] for the participants challenges and sources of stress. There were four major themes: (1) Familial Relationships, (2) Essential Work, (3) Finances, and (4) Study, as outlined in the text below. Additional subthemes, student quotes, and relevant data obtained from the questionnaires can be observed in Table 3.

### 3.4. Challenges and Sources of Stress

Theme (1). Familial Relationships: Separation from Family and Associated Disempowerment or Lack of Control.

Most participants (89%) had left family in their home country with the intention of reuniting with them within a reasonably short time, usually (pre-COVID-19) within three months of being in New Zealand.

*“…my spouse and younger daughter [were supposed to join me]. She is just 1 year [old]. We planned that they will come soon to join me but due to COVID-19 [sic] they can’t come here”.*

Such stress associated with separation stemmed from dangers in confronting family members in their home countries as 74% of participants hailed from India. Throughout much of the period in which this survey was available for completion, India led the world in daily increases in COVID-19 case numbers [44]. Participants’ commentary highlights the challenging circumstances family members faced.

*“My paternal aunt’s family has difficulty due to COVID-19, as they were infected.”*

*“Yes, my pregnant sister got COVID-19 after her visitation to gynaecologist.”*

Furthermore, family and friends were negatively impacted by the general circumstances surrounding the pandemic, as well as due to the virus itself.

*“My parents are diabetic and vulnerable to COVID infection. My grandmom is old and thus vulnerable too. It is very difficult for them to stay safe.”*

*“The situation in my home country is getting worse by the day. It is a matter of personal concern to me since my mother is a doctor who is also at high-risk due to her age.”*

Separation from family and its effect on wellbeing was reflected in participants’ reports on their coping strategies and achievements during lockdown. When asked about their main coping methods, 40 (93%) participants selected ’Contacting family abroad’, by far the most common selection. ‘Contacting peers’ was the next most frequently selected option at 53%. Similarly, when asked to select significant achievements or outcomes of the lockdown, 38 participants (88%) selected ‘Managed to connect via social media with family abroad’.

Adapting to the Increased Presence of Children: While separation from family was a commonplace challenge and linked with unhappiness or stress, there were also challenges for those who had family with them.

*“My kids are normally active, that’s why preventing them to go out is quite a challenge, they easily got bored during lockdown and increased the usage electronic devices. Adapting to new normal is quite challenging.”*

Theme (2). Essential Work.

As noted above, 29 of 43 survey participants (67%) worked in essential healthcare services during lockdown. Of these, 55% stated that they worked additional hours (i.e., on top of their regular hours); in this capacity, Immigration New Zealand allowed flexible working conditions for international students working in the health sector, making it easier for them to contribute to the COVID-19 pandemic response [45].

Fear of Contracting the COVID-19 Virus: A primary public health concern for essential workers was the potential of catching the COVID-19 virus. This concern was often expressed in terms of worry, anxiety, or being afraid.

*“I am an essential worker and worried about catching COVID-19 infection from my work colleagues.”*

Despite these concerns, the small number of participants who commented on workplace safety practices and the provision of personal protection equipment (PPE) unanimously stated that their employers had taken appropriate steps to minimise risk of infection. When asked to select significant outcomes they had achieved throughout the lockdown, 90% of essential workers stated that they had effectively protected themselves from contracting COVID-19.

*“The organization have made proper arrangements in this regard [ensuring safety]. They provide face shields, PPE gowns, face-mask and others things”.*

Workplace Intensity: Participants reported that their jobs were more demanding and draining throughout the lockdown. Much of this increased demand was characterised in terms of increased emotional labour; aged care residents/patients under the participants’ care required greater attention and protection and, in some cases, exhibited more difficult behaviours. This required increased care on the part of essential workers.

*“I was definitely afraid to work in such an atmosphere because I am a student at the end and if something was to happen to me I would not have been able to pay the costs of treatment.”*

Benefits of Being an Essential Worker: Despite the fear, emotional toll, and logistical challenges associated with essential work, participant responses also show that there were numerous benefits to being an essential worker. The first was that essential work provided a source of income.

*“Didn’t have any [financial challenges] during the pandemic… as a healthcare worker, we were allowed to work more than 20 h hence [sic] it covered all my finances”.“ As I worked in full time in pandemic time, so [sic] I didn’t face any monetary issues.”*

A second benefit was the opportunity for social interaction and the ability to stay busy. At a time when most social interactions were prohibited and many participants were unable to see their families, working offered an opportunity to maintain social connections and offered a distraction from the hardship of family separation.

*“Being busy [with work] was good allowed me to forget my issues (remembering of my kid back home).”*

Finally, there was a perceived normative benefit in having contributed to the wellbeing of others at a challenging time. Some participants linked this to a sense of responsibility or identity as a healthcare worker. Indeed, in listing their significant achievements of the lockdown, 83% of participants who worked in essential services stated that they had effectively protected patients from contracting COVID-19 and, therefore, felt a sense pride and job satisfaction.

*“It was a good experience to help elders and a rewarding job.”*

*“… I was really proud of myself and it felt great to help people when they needed me the most.”*

Theme (3). Finances.

Essential work appears to have been a leading means of financial stability. Twenty-nine (67%) participants reported carrying out essential work so that only seven participants (14%) reported accessing EIT’s hardship fund.

There was a subset of participants who did not have a job at the start of the lockdown possibly due to having only recently arrived in New Zealand, and the lockdown made finding a job difficult. Some were able to alleviate this difficulty via the Funds Transfer Scheme (FTS). The FTS is a transfer scheme sanctioned by Immigration New Zealand that allows for an easy and secure transfer of funds to students from their country of origin. Unsurprisingly, participants’ employment status at the beginning of the lockdown appears to have played an integral role in their experience of associated financial challenges.

*“It took [a] long time to get job because of COVID-19, as no face to face interview was possible. So, there was [a] delay in the process.”*

Theme (4). Study.

Surprisingly, the increased stress connected to the COVID-19 pandemic was rarely explicitly linked with struggling to perform academically. When asked to select coping strategies employed throughout the lockdown (not solely in relation to their studies), 11 (26%) participants stated that they had utilised EIT’s wellbeing support services. Furthermore, 15 (35%) participants reported utilising the assistance of course tutors. Participants typically described unique features of studying under lockdown that had made studies more difficult or less enjoyable, without linking this to their overall wellbeing. Two challenges to study imposed by the lockdown were discussed in participant commentary far more than any others. Both stem from the lack of face-to-face teaching. The first challenge was adapting to online learning. A common sentiment was that online classes took time to adjust to and were, at least initially, difficult to concentrate on for extended periods.

*“Hard to concentrate during online classes”*

The second challenge concerned a diminished student experience due to the inability to meet in person with peers or tutors and, initially, to access the range of resources that would normally be available.

*“The face to face interaction and discussion with course mates were [sic] limited. The library services were limited too.”*

### 3.5. Institutional Support and Services Provided

Despite the difficulties imposed by rapidly adapted teaching and learning practices, participants reported utilising a number of EIT’s services for either academic or pastoral support. These were reported as a major means of coping throughout the students’ lockdown. Subsequently, twenty-five participants (58%) reported utilising EIT’s library learning services, thirteen (30%) directly contacting staff employed as library learning advisors. Similarly, 19 participants (44%) took part in additional video conferencing calls with academic staff outside of class time. Institutional support targeted towards international students was also important. Nineteen participants (44%) sought support from EIT’s international student support team. Eleven participants (26%) reported having utilised their international student mentor allocated to them through EIT’s international student mentor programme.

## 4. Discussion

This is the first study from New Zealand in which both quantitative and qualitative data are presented to support the concept that international PG NHSc students studying at the tertiary education institute, EIT, experienced higher-than-optimal levels of stress caused by a combination of factors specific to this population during the COVID-19 lockdown. In particular, the dual challenges of working in essential health services whilst being separated from family had unfavourable effects on participants’ wellbeing. These challenges are not unique to international PG NHSc students [30]. However, the demographic information collected regarding essential work and separation from family suggests that such circumstances were far more prevalent among this group compared with the general population of New Zealand.

Challenges imposed by financial hardship and study pressure should not be dismissed. It is already challenging to try to adjust to the multifaceted demands and increasing pressures of tertiary education at the best of times [22,46]. However, studying at a postgraduate level in a new country away from your own family provides unique opportunities for increased stress for international students. As all courses transitioned to online learning, students initially found this challenging. However, this was mostly associated with the feelings of being in isolation and loneliness from being away from their peers and off campus, as they navigate more self-directed learning at home. However, these challenges appeared to be either less frequent or of less direct concern to the wellbeing among the population of interest as the courses progressed. These findings are of relevance as many institutions have now opted to continue online/blended teaching as a model of delivery. It is thought that online/blended courses should be interactive with innovative teaching approaches to enable student engagement [47,48].

In understanding the impact of both separation from family and working in essential services during the lockdown on participants’ wellbeing, there are a number of important aspects to consider. The thematic analysis of responses indicated that much of the stress associated with being separated from family was due to the challenges or dangers confronted by distant overseas family members rather than a lack of familial support for participants themselves. Thus, it is possible that the particular trajectory of the COVID-19 virus’ spread within India, where the majority of separated families resided, contributed both to the degree of perceived stress among the present participants and the salience of separation from families as a cause of perceived stress. In the present data, a statistically significant difference in PSS-10 scores between those hailing from India and those from elsewhere was not observed. However, the small number of participants from countries other than India means that the possibilities described above cannot be ruled out completely. Moreover, cultural values or expectations concerning familial roles and responsibilities may be at play here as a high proportion of our participants were mothers with young children who were unable to join them from their home countries [49,50]. In the present study, a direct comparison was not made between PSS-10 scores for female (88%) and male (12%) populations, with a relatively low number of participants (*n* = 42). However, Hathaway et al. [22] reported higher PSS-10 scores for 75% of the female (22.55) population compared to the 25% male (17.11) population in their study (*n* = 312). These results are in line with other studies that demonstrate higher COVID-19-related stress levels for female participants [51,52]. A cross-national study including nine countries, compared PSS-10 among university students during the COVID-19 pandemic. The highest score was found to be from Turkey (22.71 ± 6.43) with the lowest from Chechia (18.16 ± 3.99) (Ochnik et al., 2021). With a PSS-10 level of 21.7 (±7.1), our international students studying in New Zealand fall within this range. This demonstrates that higher education students in these ten countries had medium to high stress levels over the COVID-19 pandemic, making them vulnerable to mental health issues [42]. With regards to the essential health workers in the group, the social aspects and satisfaction of contributing to public health make it hard to know what net effects this might have on wellbeing generally, and perceived levels of stress in particular. The foregoing qualitative analysis highlighted that, for many participants, essential health work was initially associated with a fear of catching the virus and/or increased emotional and material labour. This is reflected by Fan et al. [53] where “high frequency and high-intensity work, including close contact with patients, produces occupational hazards and psychological stress for nurses”. This concern was expressed by participants statements in this study indicating worry, anxiety, and initially being afraid to be in the healthcare work environment, especially as they were requested to work extra hours. Despite these initial apprehensions, participants’ overall comments on workplace safety equipment and practices unanimously stated that employers had taken the appropriate steps to minimise risks of COVID-19 infection. Overall, participants reported eventually feeling reassured and safe with effective and sufficient PPE (personal protection equipment) that their employers provided. It is hoped that healthcare employers will consider these initial concerns and be better prepared for future pandemics.

Nevertheless, essential work offered a number of benefits: distraction from personal worries, social interaction, a sense of contributing to a good cause, and financial stability. Regarding the financial benefits of essential work, the qualitative analysis highlighted that those without essential work were more likely to report financial hardship during the lockdown and, therefore, ‘worried’ more. It is these students who needed to access the EIT hardship fund. Thus, essential work can be said to both present and alleviate challenges associated with increased stress. Indeed, on average, those who performed essential work had lower PSS-10 scores than those who did not (20.4 compared to 24.3); although this difference is not statistically significant, it is still above an optimal stress level [25,26,27]. Thus, the following recommendations are worth considering: Steps can be taken by employers to maximise benefits while minimising challenges. Essential work can act as a source of financial stability, yet it is often more demanding than under regular circumstances with high intensity over increased hours over the COVID-19 outbreak. The additional burden could be rewarded by employers, for example, through pay increases. Some sectors implemented such a step during New Zealand’s lockdown, with supermarket workers receiving a 10% pay increase [54]. However, this was not the same for healthcare workers over the COVID-19 lockdown.

A number of other recommendations for how tertiary institutions and employers of essential workers might be able to assist international PG NHSc students, and international students in general, for the remaining challenges of COVID-19 and in similar future scenarios can be gleaned from the data in this study. It is likely that these suggestions will need to be adapted to specific contexts. As in other sectors, tertiary education institutions were forced to adapt rapidly in the face of COVID-19 [55]. Our data suggest that students are keen to engage with their studies and their institute’s support services throughout a lockdown scenario. There was little evidence in the data to suggest that studies were deemed adverse to participants’ wellbeing. However, a small number of participants reported feeling overstretched in trying to complete studies and essential work at the same time. However, participants more commonly lamented on the lack of social interaction that usually accompanied the study [56]. Further research is necessary to explore the impact of specific social issues and public health roles of postgraduate international students and how these may have changed during the pandemic.

As the COVID-19 pandemic continues, New Zealand’s borders remain closed to new international students. However, with international vaccination rates increasing, it is hoped that these border restrictions will be lifted in the not-too-distant future and that, globally, we will once again benefit from the vast contributions that international students make to their host countries. The international PG NHSc students in this study were skilled labourers, including doctors, nurses, laboratory technicians, and pharmacists that were already qualified in the public health sector. They demonstrated that they were able to cope with studying while simultaneously contributing their skills as essential workers in hospitals and other healthcare settings over the COVID-19 lockdown, when New Zealand was in dire need. This study provides important information about levels and sources of perceived stress and how tertiary institutions and employers of essential workers might be able to assist these international students, particularly nursing and health science students, throughout the remaining challenges of COVID-19 and in similar future scenarios. Recommendations can be made and adapted to the specific contexts such as (a) Tertiary Education Institutions where the desire by international students for social interaction suggests that both personal support services and teaching methodologies employed during lockdown scenarios should be tailored to maximise real-time interpersonal interactions, albeit online, and (b) Employers of Essential Workers, since the data show that essential work provides both benefits and challenges in terms of student wellbeing. The Fear of Contracting COVID-19 was a leading concern among essential workers within our sample. Thus, consistent, identifiable, and enforceable health and safety workplace practices are likely to alleviate worry, although it is unlikely that any health and safety regimes could remove this concern completely. Furthermore, participants benefited from a sense of having made a normatively valuable contribution through their essential work in healthcare. In so far as such a perception may be linked to having successfully prevented people in care from having contracted COVID-19, workplace health and safety are important in maintaining this public health benefit of essential work. New Zealand’s healthcare system has not thus far been strained by the pandemic in the same manner as in some harder-hit countries. It is worth considering whether the perceived normative public health benefits of essential work would be maintained had there been less success in the healthcare system operating effectively to prevent COVID-19′s spread.

## 5. Limitations

There are some limitations in this study that are worth mentioning. For instance, there was a much higher proportion of female representatives (88%) as compared to males (12%). Therefore, our results do not allow for matched comparisons or may not be representative of the general international student population conducting postgraduate studies in health science or nursing in New Zealand. This research comprised a cross-sectional survey (including the PSS-10 questioner) supported by semi-structured interviews. However, there could have been a larger number of participants in the cross-sectional component of the study to avoid bias and to provide a better reflection of the general postgraduate international student population. Moreover, this is a single-center study, with a purposeful selection of participants.

## 6. Conclusions

The unique situation that international PG NHSc students at EIT found themselves in during the COVID-19 lockdown appears to have manifested itself in a variety of challenges, the combination of which are likely less common among New Zealand’s broader population. These students made a significant public health contribution as they mainly worked in the frontline of the COVID-19 outbreak. Average perceived levels of stress among participants suggest that this population experienced levels of stress during lockdown that would generally be considered higher than optimal in terms of wellbeing. In particular, separation from family and working additional hours as essential healthcare workers presented challenges to maintaining health and wellbeing. Identifying themes of key challenges and methods of coping, as we have here, offers insights for continuing to support international postgraduate health students at EIT and elsewhere. Our data show that personal and academic support services offered by tertiary institutions are utilised by students and deserve continued investment if we are to benefit from the public health contribution that these students make. Moreover, employers should recognise the increased labour (often emotional due to anxiety of contracting COVID-19) undertaken by essential workers. Key method of performing this recognition are raising remuneration and ensuring that workplace safety equipment (e.g., PPE) are sufficient and fit for certain purposes. International students who work in healthcare areas are essential for the maintenance and promotion of health in their host countries. Moreover, given the increasing public recognition of the role public health plays in pandemic responses, it is more important now than ever before to understand the wellbeing needs of these tertiary nursing and health students as public health workers so that we can best support them.

## Figures and Tables

**Table 1:** Adapted Questioner from Short Form of the Perceived Stress Scale (PSS-10).

Over the COVID-19 Lockdown Period:
How often have you been upset because of something that happened unexpectedly?
2. How often have you felt that you were unable to control the important things in your life?
3. How often have you felt nervous and ‘stressed’?
4. How often have you felt confident about your ability to handle your personal problems?
5. How often have you felt that things were going your way?
6. How often have you felt that you could not cope with all the things that you have to do?
7. How often have you been able to control irritations in your life?
8. How often have you felt that you were on top of things?
9. How often had you been angered because of things that were outside of your control?
10. How often have you felt difficulties were piling up so high that you could not overcome them?

**Table 2:** Semi-structured interview guided questions.

Discuss any changes in plans you had to bring your family (children and spouses) from your home country to New Zealand, since the COVID-19 lockdown periods and border closures?
2. What is the COVID-19 situation like with your family or friends in your home country now.
3. What are the biggest challenges you face over the COVID-19 lockdown period?
4. What are the other challenges that you face over this time?
5. How does your work as an essential worker during the COVID-19 lockdown period affect you?
6. Discuss any concerns for your personal safety as an essential worker.
7. What are your coping strategies or methods over the COVID-19 lockdown?
8. How has your institute (EIT) made things easier for you during this time?
9. Discuss any EIT services that you use to help cope over the COVID-19 lockdown?
10. Discuss any significant outcomes over the COVID-19 lockdown?

**Table 3:** Qualitative data showing the main themes, subthemes, and quotes as identified from the challenges faced by students and their sources of stress over the COVID-19 lockdown in New Zealand.

Main Themes: Challenges and Sources of Stress	Sub Themes	Student Quotes
Familial Relationships	Separation from Family and Associated Disempowerment or Lack of Control	*My children aged 12 and 8 were expected to join me or I was planning to travel back to India during the semester break in July, but neither of the plans worked since the borders were closed due to COVID-19.* *My husband and child wanted to join me very soon. It’s been 10 month[s] [since] we have applied for [a] visa, but due to COVID-19 [sic] the applications have not been [sic] processed and the borders are closed.* *It is very bad situation for us. I am living without my younger daughter and she also struggles without her mother (me).* *Yes, I want to bring my family over here. My husband is with me now. I planned to bring my children after finishing my course. With this COVID-19 [sic] issue I have no idea when I can bring my children.*
	Adapting to Increased Presence of Children	*During lockdown, being with kids at home (out of school) was challenging.* *Difficult to manage children at home.*
Essential Work	Fear of Contracting the COVID-19 Virus	*As essential work[ers] we have to work at [the] frontline, so when we go home there will be risk for infection to families.* *I was working as a cleaner in that period. So I was very afraid of work and being exposed to the sites.* *Stressing to get COVID-19 [sic] sometimes due to uncertainty about virus.* *I had some fear of using the public transport while going for my work…* *I think working as an essential worker was challenging. It’s good that I got the job but at the same time essential workers are at most risk of getting infected.*
	Workplace Intensity	*It was very tough to work during this period because it was very difficult to manage the resident when they were not allowed to see their family.* *It was more stressful as resident of the facilities are locked down in house so their behaviours was [sic] more challenging.* *At first, it was difficult to adapt to the new environment and safety measures we had to take. I was constantly worried about the safety of the residents of my workplace…* *The residents of the rest home where I work were in emotional pain during lockdown as they could not meet their family members. It was heart breaking to see them suffer mentally and emotionally. They begged us to let them meet their family but we couldn’t do that due to restrictions.* *Some of them were affected so badly that their condition started to deteriorate.*
Finances	Financial Challenges	*Difficult to find a job at first and was not able to open a bank account.* *I had to stay 2 weeks at home without pay after leaving one job to start another one.* *During the pandemic, we were unable to find part-time jobs, but FTS transfer was there so that we could manage our finances.*
Study	Study-Related Challenges	*Lack of concentration after few hours of virtual classes.* *Virtual classes were difficult to concentrate upon.* *Online classes were initially tough, but came out exciting later on.* *The live classroom delivery when compared to online delivery has twice the impact on students.so if the alert level is reduced, I always prefer to have live classes.* *Did online, but miss physical gathering [sic] and classmates.* *Being unable to seek in-person support from the tutors and library services was troublesome but got on track in a few weeks’ time.* *Lack of discussion about the topic with tutors and friends.* *Limited or no access to library due to COVID.* *Difficulties in assessments as lib [rary] and college face to face facilities were not working.*

## Data Availability

Anonymized data are available upon request from the corresponding author.
